# Comparison of the Laryngeal View during Tracheal Intubation Using Airtraq and Macintosh Laryngoscopes by Unskillful Anesthesiology Residents: A Clinical Study

**DOI:** 10.1155/2011/301057

**Published:** 2011-11-17

**Authors:** Carlos Ferrando, Gerardo Aguilar, F. Javier Belda

**Affiliations:** Departamento de Anestesiología y Cuidados Críticos, Hospital Clínico Universitario de Valencia, Avenida Blasco Ibáñez No.17, 46010 Valencia, Spain

## Abstract

*Background and Objective*. The Airtraq laryngoscope (Prodol Meditec, Vizcaya, Spain) is a novel tracheal intubation device. Studies, performed until now, have compared the Airtraq with the Macintosh laryngoscope, concluding that it reduces the intubation times and increase the success rate at first intubation attempt, decreasing the Cormack-Lehane score. The aim of the study was to evaluate if, in unskillful anesthesiology residents during the laryngoscopy, the Airtraq compared with the Macintosh laryngoscope improves the laryngeal view, decreasing the Cormack-Lehane score. *Methods*. A prospective, randomized, crossed-over trial was carried out on 60 patients. Each one of the patients were intubated using both devices by unskillful (less than two hundred intubations with the Macintosh laryngoscope and 10 intubations using the Airtraq) anesthesiology residents. The Cormack-Lehane score, the success rate at first intubation attempt, and the laryngoscopy and intubation times were compared. *Results*. The Airtraq significantly decreased the Cormack-Lehane score (*P* = 0.04). On the other hand, there were no differences in times of laryngoscopy (*P* = 0.645; IC 95% 3.1, +4.8) and intubation (*P* = 0.62; C95%  −6.1, +10.0) between the two devices. No relevant complications were found during the maneuvers of intubation using both devices. *Conclusions*. The Airtraq is a useful laryngoscope in unskillful anesthesiology residents improving the laryngeal view and, therefore, facilitating the tracheal intubation.

## 1. Introduction

Nowadays, the direct conventional laryngoscopy is usually performed with the Macintosh laryngoscope, considered the gold standard for tracheal intubation since the 1940s [1]. At the present time, as an alternative to the Macintosh laryngoscope, there are several laryngoscopes and other optical devices used for difficult tracheal intubation, which are very well known by specialists and which require an appropriate training. It must be emphasized that the failure of tracheal intubation, in spite of the advances in the laryngoscope devices, remains the most common cause of morbility and mortality both in anesthesia and in emergency situations [2, 3].

The difficulty of the laryngoscopy mainly changes according to the patient characteristics, and there are several classifications and patient characteristics that are taken into account in order to foresee a difficult airway [4, 5]. The absence of an index that could really predict the existence of a difficult airway means that many difficult intubations are recognized only after the anesthetic induction [5, 6]. 

The Airtraq (Prodol Meditec, Vizcaya, Spain) was developed in 2005. In contrary to the other laryngoscopes, it has been designed for visualising the glottis without alignment of the oral, pharyngeal, and tracheal axes. The Airtraq has two channels, the guide channel, where the tube is placed, and the optical channel, which ends in a distal lens that enables the user to visualize the glottis, having a battery at the top. The optical vision is received through a series of 5 lens and 2 mirrors placed in the interior of the device. It also contains an antifogging system [7]. The tracheal tube does not obstruct the view during intubation, and any tracheal tube of any type and size can be used. The laryngoscopic technique is very simple: with the head in a neutral position, the blade is inserted into the midline of the mouth over the tongue, and the tip of the blade is placed in the vallecula [8]. This device is disposable, providing, therefore, unarguable advantages when avoiding disease transmission [9]. 

To date, there are no clinical studies comparing the laryngoscopy using the Airtraq and Macintosh laryngoscopes by unskillful physicians. Classical difficult intubation predictors (Mallampati, thyromental distance, mouth opening) do not predict a difficult laryngoscopic view when we use Airtraq optical laryngoscope. The utility of these predictors is uncertain when they are applied to videolaryngoscopic's use. There is only a predictive test's work for the Glidescope videolaryngoscope that shows a decrease of the specificity and sensitivity of the El-Ganzouri multivariate risk index when they use videolaryngoscopy [10]. 

For this reason, we designed this study with the aim to evaluate if the laryngoscopy, performed by unskillful anesthesiology residents using the Airtraq compared to the Macintosh laryngoscope, decreases the Cormack-Lehane score, and improves the laryngeal view. Additionally, we compared the success rate at first intubation attempt, the intubation times, and the complications with both techniques.

## 2. Methods

A prospective, randomized, crossed-over trial was carried out. After the Ethics Committee approval and when informed consent was obtained, we studied patients scheduled for any kind of surgery who required tracheal intubation. Exclusion criteria were patients who could require rapid sequence induction, ASA physical status 4, age under 18 yr, and an interincisor distance less than 3 cm. The latter were excluded in order to avoid any complication when introducing the Airtraq, due to its diameter.

Anesthesiology unskillful residents of our department conducted the laryngoscopies. Previously to the beginning of the study, they had performed in patients less than two hundred intubations with the Macintosh laryngoscope and 10 intubations using the Airtraq.

Once patients were on the operating room, they were preoxygenated with FiO_2_ at 100% during three minutes. After this, a standard anesthetic induction was performed using intravenous anesthetics: fentanyl (1.5 *μ*g Kg^−1^), propofol (2,5 mg kg^−1^), cisatracurium (0,15 mg kg^−1^). A routine monitoring was used during the study (electrocardiogram, noninvasive blood pressure, pulse oximetry, capnography, and neuromuscular block monitoring). A chronometer was used to collect the different laryngoscopic and intubation times. There were two groups (M and A). The patients were randomly assigned to each group by random numbers (EXCEL Random Generator, Microsoft), and all of them underwent two laryngoscopies in sniffing position. In the group M, the laryngoscopy was first made with the Macintosh, and in the group A it was first made with the Airtraq. The time interval between the two laryngoscopies was two minutes. The tracheal intubation was carried out with the device used in the second laryngoscopy. All the patients were intubated with a 7.5 mm internal diameter tracheal tube. 

During the following five minutes after the laryngoscopy, the lung was ventilated with volume control mode (8 ml Kg^−1^), and the anesthesia was maintained with sevoflurane (1.25–1.75% of end tidal concentration) with a mixture of 60% nitrous and oxygen. The maintenance of the anesthesia was carried out according to each patient's requirements. 

Before conducting the anesthesia, a series of demographic data (age, gender, body mass index, and ASA Physical status) was collected. We performed the evaluation of the airway including tests for predicting difficult tracheal intubation (Mallampati score, thyromental distance, and cervical movement) as well as other factors that could affect the laryngoscopy such as beard, dental prostheses, or obstructive sleep apnea syndrome (OSAS). We recorded in all laryngoscopies: the Cormack-Lehane score, the laryngoscopy time (time elapsed from the introduction of the laryngoscope into the dental arch until the glottis was visualized), and the intubation time (time elapsed from the introduction of the laryngoscope into the dental arch until the investigator confirmed, by lung auscultation, the tracheal intubation). Other data recorded for evaluating the simplicity of one or another technique was the need to reposition the patient, the need to perform the back, up, right, pressure (BURP) maneuver, and the number of unsuccessful attempts until the tracheal intubation was achieved. An unsuccessful intubation attempt was defined as the nontracheal intubation in 90 sec or a patient's desaturation (SatO_2_ < 90%).

A minimum sample size of 59 laryngoscopies with each device was calculated to detect a reduction of 1 grade in the Cormack-Lehane score with a power of 90% and a significance of 95%.

All data were analyzed by SPSS statistical software (version 15.0, SPSS Inc., Chicago, Ill, USA). Data of the Cormack-Lehane score, number of attempts, need for repositioning of the patient, esophageal intubations, dental trauma, and failed tracheal intubation were analyzed using Mann-Whitney *U*-test. The data of the laryngoscopy and intubation times were compared using the Student *t*-test. The continuous data are shown as mean ± standard deviation, and categorical data are presented as absolute numbers and percentages.

## 3. Results

A total of 60 patients were included into the study. 120 laryngoscopies were performed, 60 using the Airtraq and 60 using the Macintosh. No patients refused consent to participate in the study.  Table 1 shows the demographic data of the selected population. Descriptive data of previous evaluation of the airway is given in Table 2.

Of the 120 laryngoscopies performed using the Airtraq device and Macintosh laryngoscope, the Cormack-Lehane grade obtained is shown in Table 3. Cormack-Lehane was statistically significantly lower with the Airtraq device (*P* = 0.04). The optimization maneuver required for both devices is shown in Table 4. Among the patients of the Macintosh group, 24 were successfully intubated on the first attempt; 6 were treated with a repositioning maneuver in order to achieve the proper alignment of the airway and; therefore, a second attempt was required (Table 4). The patients of the Airtraq group were all successfully intubated on the first attempt except for one. The failure was produced because the light of the device went out. This patient was intubated using the Macintosh laryngoscope (Table 4). There was no esophageal intubation, regardless of the laryngoscope used. In spite of the unskillful anesthesiology resident, no dental trauma or any other complication not indicated above occurred in any of the 60 laryngoscopies performed.

The differences in time elapsed for laryngoscopy and intubation were not significantly different between the techniques, as shown in Table 5.

## 4. Discussion

Once the analysis of the results of this study is carried out in the 120 laryngoscopies performed by unskillful anesthesiology residents, our findings show that there is a significant reduction (*P* = 0.04) of the Cormack-Lehane score when the laryngoscopy is performed using the Airtraq laryngoscope. Despite the fact that the Cormack-Lehane score was designed for the direct conventional laryngoscopy, in our study it was useful to predict the laryngoscopies outcome using both devices. 

Most of the studies that compare Airtraq with other devices had been conducted by experienced personnel (more than 500 tracheal intubation) in situations of difficult intubations or by novice personnel using manikins [7, 8, 11–17]. There is only one study where both devices (Airtraq and Macintosh) performing intubations are compared during the routine management of the airway [2]. In this study there was a reduction of the Cormack-Lehane score as the one we have observed, but in this case the study was carried out by experienced personnel. 

As shown in Table 4, no significant differences in the number of attempts or in the appearance of complications related to the laryngoscopy were found. However, there were significant differences in the performance of airway optimization maneuvers. Due to the characteristics of the device, there was no need to perform any of these maneuvers when using the Airtraq. The results of this study are similar with those of other papers [8, 11–17] which show that with the Airtraq it is not necessary to carry out maneuvers to optimize the patients airway, and; therefore, a short number of dental trauma occur. 

In contrast to the results obtained in this study, the majority conclude that with the Airtraq a statistically significant increase in the successful intubations both in normal and difficult airway is obtained. Most of these studies are performed in difficult airway. These differences probably occur because the Airtraq facilitates the tracheal intubation. In our study any differences were found in normal airway unlike other papers above mentioned. The reason is that our group is more experienced using the Macintosh laryngoscope rather than the Airtraq.

One of the aims of the study was to determine if there were differences both in laryngoscope and intubation times. Table 5 shows that the laryngoscopy and intubation times are longer using the Airtraq, although the difference is not significant, as happened in previous studies [2, 8, 11–14]. However, these results differ from other studies where the times were significantly shorter. These variations in time with regard to previously published papers are difficult to evaluate due to the influence of several factors such as the experience of the person carrying out the laryngoscopy, the degree of difficulty of the airway, or if the maneuver is performed on a patient or on a manikin. 

 There are some limitations in our study. Firstly, it cannot be a double-blind study because the observer collecting the data is present at the operating room. Another limitation of the study is that the group of patients with an interincision distance lower than 3 cm had to be excluded in order to avoid difficulties when introducing the Airtraq. However, no patient was excluded for this reason. 

Other authors [2, 8, 11–19] have found, as in our study, that the Airtraq appears to have more advantages over the Macintosh laryngoscope. The Airtraq decreases the grade of Cormack-Lehane's score with regard to the Macintosh in laryngoscopies performed by unskillful anesthesiology residents. At the same time, it decreases the need to conduct airway optimization maneuvers in order to align the axes. Cormack and Lehane's classification was designed to evaluate the degree of difficulty for the intubation by direct laryngoscopy describing the anatomical structures visualised. Then, it rises an important question: is this classification really valid when we realise an intubation with a videolaryngoscopy or an optical laryngoscope without alignment of the three airway axes? It is clear that like comparative system with the direct laryngoscopy is the only classification that has been available. In fact, some authors have described a new four degrees classification for the laryngoscopic vision with Airtraq optical laryngoscope [20, 21]. Moreover, the laryngoscopic technique using Airtraq does not increase the intubation and laryngoscope times as well as it does not increase the number of complications or hemodynamic stimulation.

Therefore, we conclude that the Airtraq can be an alternative to the Macintosh laryngoscope when used by unskillful anesthesiology residents during scheduled surgeries, improving the laryngeal view and, therefore, facilitating the tracheal intubation. Additionally, this study highlights the indispensable role of Airtraq in the hands of inexperienced staff designated to conduct tracheal intubation as part of their duties in emergency scenarios [2, 7, 9, 12, 14].

## Figures and Tables

**Table 1:** Demographic data. Data are shown as absolute number of patients or mean (± SD). BMI: body mass index.

Gender (m : f)	41 : 19
Age	46 (24)
BMI	
<25	41 (68.3)
25–30	14 (23.3)
>25	5 (8.3)
ASA physical status classification system	
1	39 (65)
2	16 (26)
3	5 (8)
4	0 (0)

**Table 2:** Airway evaluation. Data are shown as absolute number of patients (percentage). OSAS: obstructive sleep apnea syndrome.

Mallampati's score	
1	29 (48)
2	20 (33)
3	10 (16)
4	1 (1.6)

Thyromental distance	
<6 cm	10 (17)
>6 cm	50 (83)

Dental prostheses	
Yes	19 (32)
No	41 (68)

Cervical flection	
<45°	3 (5)
45°–90°	18 (30)
>90°	39 (65)

Beard	
Yes	5 (8)
No	45 (92)

OSAS	
Yes	6 (10)
No	54 (90)

**Table 3:** Cormack-Lehane's score in the patients using the Airtraq and the Macintosh laryngoscopes. Data are shown as absolute numbers of patients (percentage).

Cormack-Lehane's grade	Airtraq (*n* = 60)	Macintosh (*n* = 60)
1	56 (93,3)	34 (56.6)
2	3 (5)	16 (26,6)
3		9 (15)
4	1 (1,6)	1 (1,6)

**Table 4:** Airway optimization maneuvers in the patients using the Airtraq and the Macintosh laryngoscopes. Data are shown as absolute numbers (percentage). BURP: back, up, right, pressure.

	Macintosh (*n* = 60)	Airtraq (*n* = 60)
Repositioning of the patient	15 (25)	0 (0)
BURP	21 (35)	0 (0)
Number of attempts *n* = 30		
1	24 (80)	29 (96.6)
2	6 (20)	0 (0)
>2	0 (0)	0 (0)
Failed tracheal intubation	0 (0)	1 (3.3)

**Table 5:** Complications in the patients using the Airtraq and the Macintosh laryngoscopes. Data are shown as absolute numbers (percentage). *Laryngoscope light failure.

Complications	Macintosh	Airtraq
Esophageal intubation *n* = 60	0 (0)	0 (0)
Dental injury *n* = 120	0 (0)	0 (0)
Other complications *n* = 120	0 (0)	1 (0.83)*
